# Screen time is associated with adiposity and insulin resistance in children

**DOI:** 10.1136/archdischild-2016-312016

**Published:** 2017-03-13

**Authors:** Claire M Nightingale, Alicja R Rudnicka, Angela S Donin, Naveed Sattar, Derek G Cook, Peter H Whincup, Christopher G Owen

**Affiliations:** 1 Population Health Research Institute, St George's, University of London, London, UK; 2 Institute of Cardiovascular & Medical Sciences, University of Glasgow, Glasgow, UK

**Keywords:** Childhood, Type 2 diabetes, Adiposity

## Abstract

**Background:**

Higher screen time is associated with type 2 diabetes (T2D) risk in adults, but the association with T2D risk markers in children is unclear. We examined associations between self-reported screen time and T2D risk markers in children.

**Methods:**

Survey of 4495 children aged 9–10 years who had fasting cardiometabolic risk marker assessments, anthropometry measurements and reported daily screen time; objective physical activity was measured in a subset of 2031 children.

**Results:**

Compared with an hour or less screen time daily, those reporting screen time over 3 hours had higher ponderal index (1.9%, 95% CI 0.5% to 3.4%), skinfold thickness (4.5%, 0.2% to 8.8%), fat mass index (3.3%, 0.0% to 6.7%), leptin (9.2%, 1.1% to 18.0%) and insulin resistance (10.5%, 4.9% to 16.4%); associations with glucose, HbA_1c_, physical activity and cardiovascular risk markers were weak or absent. Associations with insulin resistance remained after adjustment for adiposity, socioeconomic markers and physical activity.

**Conclusions:**

Strong graded associations between screen time, adiposity and insulin resistance suggest that reducing screen time could facilitate early T2D prevention. While these observations are of considerable public health interest, evidence from randomised controlled trials is needed to suggest causality.

**What is already known on this topic?**

- Increased screen time is prospectively associated with adiposity and type 2 diabetes in adults.
- Evidence suggests graded associations between screen time and adiposity in children.

**What this study adds?**

- This study demonstrated strong graded positive associations between screen time, adiposity and risk markers for type 2 diabetes (particularly insulin resistance) in children.
- The associations between screen time and insulin resistance markers were largely independent of socioeconomic status, pubertal status, objectively measured physical activity and adiposity.

## Introduction

The global prevalence of type 2 diabetes (T2D), overweight and obesity has been increasing in adults as well as adolescents and children.^1^ ^2^ Awareness of the early determinants of adiposity and T2D risk in young people may be important for reducing risks of T2D and obesity across the life course. The health effects of activities that encourage sedentary behaviour, such as time spent watching television, using computers or games consoles (together referred to as ‘screen time’) have been a particular focus. Increased screen time has been prospectively associated with adiposity^3^ and T2D risk in adults.^4^ Studies have shown graded positive associations between prolonged screen time and adiposity in childhood.^5^ ^6^ However, less is known about the effects of prolonged screen time on T2D risk markers in childhood, particularly insulin resistance (IR) and glycaemia. Such associations could be of public health importance in providing evidence-based recommendations for healthy screen-time duration, especially as recent trends suggest that screen time is increasing in childhood.^7^ We therefore examined the association between self-reported daily screen time and risk markers for T2D and cardiovascular disease, in a large-scale multi-ethnic population-based study of children aged 9–10 years.

## Methods

The Child Heart and Health Study in England (CHASE) was a cross-sectional survey of cardiovascular health in UK schoolchildren of white European, black African-Caribbean and South Asian origin aged 9–10 years carried out in a sample of 200 primary schools in London, Birmingham and Leicester; full details of the study have been reported elsewhere.^8–10^ Ethical approval for the study was obtained from the relevant multicentre ethics committee. A single survey team carried out all measurements between October 2004 and February 2007. Children's ethnic origin was based on the ethnicity of both parents or (where not available) the parentally defined ethnicity of the child; in a small proportion of children where this was not available (1%), child information on the place of birth of parents and grandparents was used to define ethnicity. Ethnic group was classified as ‘white European’, which includes those of white European including white British ethnicity; ‘black African-Caribbean’, which includes black African, black Caribbean and black other; ‘South Asian’, which includes Indian, Pakistani, Bangladeshi and South Asian other; those of mixed or other ethnic origins were included in an ‘other ethnic groups’ category. Socioeconomic status was based on self-reported parental occupation (highest of mother or father) and coded using the National Statistics Socio-economic Classification (NS-SEC).^11^

Height was measured to the last complete millimetre using a portable stadiometer (Chasmors, London, UK) and weight was measured using an electronic digital scale (Tanita, Tokyo, Japan). Ponderal index was calculated as a weight divided by height cubed (kg/m^3^), providing a weight for height measure which was largely independent of height in the study population. Right-sided skinfolds were measured at four locations (biceps, triceps, subscapular, suprailiac) and summed. Hand-to-foot bioimpedance was measured using the Bodystat 1500 bioimpedance monitor (Bodystat, Isle of Man, UK); fat mass was derived using ethnic and sex-specific validated equations^12^ and presented as a fat mass index (fat mass/height^5^), which was independent of height. Blood samples were collected after an overnight fast. HbA_1c_ was analysed in whole blood by ion exchange high-performance liquid chromatography. Plasma glucose was measured using the hexokinase method; triglyceride, high-density lipoprotein (HDL) cholesterol and total cholesterol were measured in serum using an Olympus autoanalyser. Insulin was measured in serum using an ELISA method,^13^ C reactive protein (CRP) using ultra-sensitive nephelometry and leptin using a radioimmunoassay. Homeostasis model assessment (HOMA) equations provided an estimate of IR.^14^ Seated blood pressure was measured twice in the right arm after 5 min rest using an Omron 907 blood pressure monitor and adjusted for appropriate cuff size^15^; average systolic and diastolic blood pressures were calculated from the two readings. In a subset of children in the last 80 schools studied, objective physical activity counts and time spent in moderate-to-vigorous physical activity (MVPA) were measured using a waist-worn accelerometer (ActiGraph GT1M, ActiGraph, Pensacola, Florida, USA).^9^ Pubertal status was measured in girls only, using the Tanner breast development score.^16^ Child questionnaires administered on the same day that physical measurements were taken asked, ‘How many hours each day do you spend watching television or video and playing computer games?’ and children were asked to tick one of the following options: ‘none; an hour or less; one to two hours; two to three hours or more than three hours’.

Outcomes following approximately log-normal distributions were log-transformed; geometric means and percentage differences are presented for log-transformed variables. We compared differences in outcomes by self-reported screen time category using those that reported ‘an hour or less of screen-time’ as a reference group and tested for a linear trend using multilevel linear regression models adjusted for sex, age (in quartiles) ethnicity and month as fixed effects. The intraclass correlation (ICC) for school-level clustering was between 0.02 and 0.09 for all cardiometabolic risk markers and physical activity counts and was higher for time spent in MVPA (ICC=0.32); hence, it was important to adjust for school as a random effect to account for clustering of children within schools. The effect of adjustment for socioeconomic status (NS-SEC), pubertal status (girls only) and physical activity was assessed because these are potential confounders of the association between screen time and diabetic risk markers. Further adjustment for adiposity was also made, acknowledging that this may be a potential mediator. We examined whether associations between screen time and outcome variables were consistent in boys and girls and in different ethnic groups by testing for an interaction between screen time and sex or ethnic group.

## Results

Of the 8641 children who were invited, 5887 (68%) took part in the study. Of these, 4884 (83%) provided a fasting blood sample. Analyses focused on 4495 children (2337 girls and 2158 boys), average age 9.9 years (95% reference range 9.2 to 10.7 years), who answered the question on daily screen time, had full anthropometric measurements and a fasting blood sample. Of these, 2031 children also had measurements of physical activity using accelerometery. Participant characteristics are shown in online supplementary table S1, including mean age of participants, gender and ethnic balance, head of household socioeconomic classification and month of measurement. Overall 4% of the study population reported no screen time, 37% reported an hour or less, 28% reported 1–2 hours, 13% reported 2–3 hours and 18% reported more than 3 hours of screen time. A larger proportion of boys (22%) than girls (14%) reported more than 3 hours of daily screen time. A higher proportion of black African-Caribbeans (23%) reported more than 3 hours of daily screen time compared with white Europeans (16%) and South Asians (16%). Adiposity and cardiometabolic risk markers are shown by sex and overall in table 1. In general, girls had higher levels of adiposity markers, leptin, fasting insulin, IR, triglyceride, CRP and lower levels of fasting glucose and HDL cholesterol than boys; girls had lower levels of physical activity counts and time spent in MVPA than boys. There were trends between screen time and ponderal index, sum of skinfolds and fat mass index (table 2). Levels of these adiposity variables were higher among children who reported more than 3 hours of screen time compared with those who reported an hour or less of screen time. There was a strong trend between screen time, leptin, fasting insulin and HOMA-IR (table 2). Children who reported more than 3 hours of screen time had higher levels of leptin (9.2%, 95% CI 1.1% to 18.0%), insulin (10.7%, 5.1% to 16.7%) and HOMA-IR (10.5%, 4.9% to 16.4%) compared with those who reported an hour or less. Adjustment for fat mass index reduced effect sizes for insulin and IR by approximately one-quarter (7.9%, 95% CI 2.9% to 13.1%; 7.7%, 2.8% to 13.0% respectively). There was a borderline significant trend between screen time and triglycerides and physical activity counts and time spent in MVPA. There was no formal evidence of a trend between screen time and HbA_1c_, fasting glucose and other cardiovascular risk factors, including lipids and blood pressure (even with further adjustment for height). Associations between screen time and fasting insulin, IR, ponderal index, sum of skinfolds and fat mass index were not appreciably affected by adjustment for socioeconomic status or physical activity measures in a subset (ie, the magnitude of associations were similar and statistically significant associations remained significant). Adjustment for pubertal status in girls slightly weakened associations for IR markers though associations for fasting insulin and HOMA-IR remained statistically significant; associations for adiposity markers were attenuated though the association with ponderal index remained statistically significant. There was no evidence that associations differed in boys and girls and between ethnic groups (all tests for interaction p≥0.05, data available from authors).

**Table 1:** Adiposity and cardiometabolic risk markers: overall and by sex

	Boys (n=2158)	Girls (n=2337)	All participants (n=4495)
Geometric mean (95% CI)			
Ponderal index (kg/m^3^)	13.0 (13.0 to13.1)	13.2 (13.1 to 13.3)	13.1 (13.1 to 13.2)
Sum of skinfolds (mm)	36.7 (35.9 to 37.6)	45.7 (44.7 to 46.7)	41.1 (40.4 to 41.9)
Fat mass index (kg/m^5^)	1.91 (1.87 to 1.94)	2.17 (2.13 to 2.21)	2.04 (2.01 to 2.07)
HbA_1c_ (%)	5.24 (5.22 to 5.26)	5.25 (5.23 to 5.26)	5.24 (5.23 to 5.26)
Glucose (mmol/L)	4.56 (4.54 to 4.58)	4.47 (4.45 to 4.49)	4.51 (4.50 to 4.53)
Insulin (mU/L)	6.46 (6.23 to 6.69)	8.14 (7.86 to 8.43)	7.28 (7.07 to 7.51)
HOMA-IR	0.82 (0.79 to 0.85)	1.02 (0.99 to 1.06)	0.92 (0.89 to 0.95)
Leptin (ng/mL)	7.15 (6.83 to 7.48)	11.49 (11.00 to 12.00)	9.15 (8.83 to 9.48)
C reactive protein (mg/L)	0.44 (0.42 to 0.47)	0.57 (0.54 to 0.60)	0.50 (0.48 to 0.53)
Triglycerides (mmol/L)	0.76 (0.74 to 0.77)	0.85 (0.84 to 0.87)	0.81 (0.79 to 0.82)
HDL cholesterol (mmol/L)	1.53 (1.51 to 1.54)	1.44 (1.42 to 1.45)	1.48 (1.47 to 1.49)
LDL cholesterol (mmol/L)	2.60 (2.57 to 2.64)	2.60 (2.57 to 2.63)	2.60 (2.58 to 2.63)
Total cholesterol (mmol/L)	4.53 (4.49 to 4.56)	4.47 (4.44 to 4.50)	4.50 (4.47 to 4.52)
Mean (95% CI)			
Systolic BP (mm Hg)	105.2 (104.6 to 105.7)	104.2 (103.7 to 104.8)	104.7 (104.2 to 105.2)
Diastolic BP (mm Hg)	62.8 (62.3 to 63.3)	63.0 (62.5 to 63.5)	62.9 (62.4 to 63.3)
Daily counts*	437 416 (429 717 to 445 114)	362 857 (355 382 to 370 332)	397 973 (391 604 to 404 342)
Daily time spent in MVPA (mins)*	78.4 (75.9 to 80.9)	61.6 (59.1 to 64.1)	69.5 (67.1 to 71.9)

Means (geometric means) adjusted for sex (all participants), age (quartiles), ethnic group, month and a random effect to allow for clustering within schools.

*Based on 2031 participants with data for daily counts and time spent in MVPA, the numbers for each screen time group were as follows: none=80, an hour or less=729, 1–2 hours=593, 2–3 hours=270, more than 3 hours=359.

Geometric means are presented for log-transformed variables.

BP, blood pressure; HDL, high-density lipoprotein; HOMA, homeostasis model assessment; IR, insulin resistance; LDL, low-density lipoprotein; MVPA, moderate-to-vigorous physical activity.

**Table 2:** Associations between self-reported screen time (television, video and computer games) and adiposity and cardiometabolic risk markers

	None (n=180)	An hour or less (n=1664)	1–2 hours (n=1268)	2–3 hours (n=575)	More than three hours (n=808)	p (linear trend)
% difference compared with ‘An hour or less self-reported screen time’ as the reference group (95% CI), p (difference)						
Ponderal index (kg/m^3^)	−1.2 (−3.7 to 1.3)	0	1.5 (0.3 to 2.8)	1.0 (−0.6 to 2.6)	1.9 (0.5 to 3.4)	0.003
Sum of skinfolds (mm)	−5.7 (−12.4 to 1.6)	0	4.6 (1.0 to 8.4)	2.3 (−2.3 to 7.1)	4.5 (0.2 to 8.8)	0.01
Fat mass index (kg/m^5^)	−5.9 (−11.2 to −0.2)	0	2.8 (0.0 to 5.7)	1.6 (−2.0 to 5.3)	3.3 (0.0 to 6.7)	0.01
HbA_1c_ (%)	0.4 (−0.6 to 1.3)	0	0.3 (−0.2 to 0.7)	0.6 (0.0 to 1.1)	0.0 (−0.5 to 0.5)	0.68
Glucose (mmol/L)	−0.4 (−1.6 to 0.8)	0	0.5 (−0.1 to 1.0)	0.1 (−0.6 to 0.9)	0.2 (−0.5 to 0.8)	0.40
Insulin (mU/L)	3.1 (−6.2 to 13.3)	0	9.0 (4.2 to 14.0)	4.9 (−1.0 to 11.2)	10.7 (5.1 to 16.7)	<0.001
HOMA-IR	3.2 (−6.1 to 13.3)	0	8.9 (4.2 to 13.9)	5.3 (−0.6 to 11.6)	10.5 (4.9 to 16.4)	<0.001
Leptin (ng/mL)	−9.0 (−20.9 to 4.6)	0	10.9 (3.8 to 18.5)	9.3 (0.3 to 19.1)	9.2 (1.1 to 18.0)	0.002
C reactive protein (mg/L)	−23.5 (−37.4 to −6.6)	0	1.8 (−7.4 to 12.0)	0.8 (−10.9 to 14.1)	0.9 (−9.7 to 12.8)	0.26
Triglycerides (mmol/L)	0.7 (−5.0 to 6.7)	0	3.2 (0.4 to 6.1)	2.2 (−1.4 to 5.9)	3.3 (0.0 to 6.7)	0.05
HDL cholesterol (mmol/L)	−0.7 (−3.7 to 2.4)	0	−1.1 (−2.5 to 0.4)	0.1 (−1.8 to 2.0)	−1.6 (−3.3 to 0.1)	0.17
LDL cholesterol (mmol/L)	−2.2 (−5.8 to 1.6)	0	−1.1 (−2.9 to 0.7)	−2.5 (−4.7 to −0.1)	−2.0 (−4.1 to 0.1)	0.06
Total cholesterol (mmol/L)	−1.3 (−3.8 to 1.3)	0	−0.6 (−1.8 to 0.6)	−1.1 (−2.6 to 0.5)	−1.4 (−2.8 to 0.1)	0.10
Difference compared with ‘An hour or less self-reported screen time’ as the reference group (95% CI), p (difference)						
Systolic BP (mm Hg)	0.3 (−1.3 to 1.9)	0	0.2 (−0.6 to 0.9)	−0.2 (−1.2 to 0.7)	−0.5 (−1.4 to 0.4)	0.24
Diastolic BP (mm Hg)	0.0 (−1.4 to 1.4)	0	0.0 (−0.7 to 0.7)	−0.2 (−1.0 to 0.7)	0.0 (−0.8 to 0.8)	0.85
Daily counts*	11 286 (−10 753 to 33 325)	0	−4720 (−15 012 to 5572)	−13 490 (−26 724 to −256)	−6637 (−18 815 to 5541)	0.05
Daily time spent in MVPA (mins)*	2.0 (−2.3 to 6.3)	0	−0.3 (−2.3 to 1.7)	−2.6 (−5.2 to −0.1)	−1.2 (−3.6 to 1.2)	0.06

Differences (% differences) adjusted for sex, age (quartiles), ethnic group, month and a random effect to allow for clustering within schools.

Percentage differences are presented for log-transformed variables.

*Based on 2031 participants with data for daily counts and daily time spent in MVPA, the numbers for each screen time group were as follows: none=80, an hour or less=729, 1–2 hours=593, 2–3 hours=270, more than 3 hours=359.

BP, blood pressure; HDL, high-density lipoprotein; HOMA, homeostasis model assessment; IR, insulin resistance; LDL, low-density lipoprotein; MVPA, moderate to vigorous physical activity.

10.1136/archdischild-2016-312016.supp1 supplementary table Study sample characteristics in 4495 participants

## Discussion

The present study showed an association between screen time and measures of adiposity, which has previously been observed in prospective studies of children.^5^ We extend these observations by demonstrating strong graded associations with T2D risk factors, particularly IR (although not glucose or HbA_1c_). The association for IR was independent of socioeconomic markers, pubertal status and objectively measured physical activity levels. The association with IR was substantially independent of adiposity (fat mass index).

Strengths of the present study include the large sample size, measurement of key T2D risk markers including IR^17^ ^18^ and the assessment of potential confounders including socioeconomic markers, pubertal status and physical activity. The children in the study were asked about the amount of time spent watching television, video or playing computer games which was appropriate to capture ‘screen-time’ when the study was conducted in 2004–2007. Studies in current settings would also need to take account of the use of more recently introduced electronic devices, for example, electronic tablets and smartphones, also potentially related to sedentary behaviour, which are now more widely used by children. Pubertal status was only assessed in girls in the present study, as evidence suggests that age of pubertal onset in boys is substantially higher than that in our study population.^19–21^ The CHASE study was powered to detect small (∼0.2 SD) ethnic differences in risk markers presented here; however, we have been able to estimate associations between screen time and adiposity and cardiometabolic risk markers with narrow CIs, suggesting a high level of precision. Although the study response rate was modest, the characteristics of participants who provided a fasting blood sample were mostly similar to participants who did not.^10^ The participants in this study were recruited from three UK cities (London, Birmingham and Leicester) which together account for two-thirds of South Asians and black African-Caribbeans in the UK. The study sample is therefore likely to be representative of these ethnic minority populations; it may be less representative of white Europeans, as they were recruited from the same schools as the ethnic minority participants. However, the representativeness is unlikely to have biased the association between screen time and IR.^22^

While the present findings are of considerable potential public health interest, evidence from randomised controlled trials is needed to establish causality. Intervention studies in children showing decreased body size associated with reduced screen time are supportive of a causal effect,^23^ ^24^ although causal associations between screen viewing and early T2D risk factors remain to be established. Future studies could illuminate the causal pathways by which screen time manifests itself such as diet and lack of breaks in sedentary behaviour as well as decreased physical activity. Recommendations from the American Academy of Pediatrics (AAP) previously suggested that children should limit daily screen-time to <2 hours,^25^ although the most recent AAP guidance did not propose a time-limit, instead suggesting that parents should place consistent limits on hours per day of media use,^26^ proposing a more pragmatic interpretation given the pervasive use of electronic devices.^7^ However, such limitations, which may be beneficial for other aspects of health, must not overlook the underlying sedentary nature of screen-related activities and their potential impact on metabolic health. Our findings suggest that reducing screen time may be beneficial in reducing T2D risk factors, in both boys and girls and in different ethnic groups from an early age. This is particularly relevant, given rising levels of T2D, the early emergence of T2D risk^1^ and recent trends suggesting that screen-time-related activities are increasing in childhood^7^ and may pattern screen-related behaviours in later life.^27^
