# Supplementary material for: Screen time is associated with adiposity and insulin resistance in children
Source: Arch Dis Child. 2017 Mar 13;102(7):612–6. doi: 10.1136/archdischild-2016-312016 (PMC5519944; doi:10.1136/archdischild-2016-312016)
Supplement: supplementary table — Study sample characteristics in 4495 participants [file archdischild-2016-312016supp001.pdf]

Supplementary Table 1: Study sample characteristics in 4495 participants

Study sample characteristics	All participants	
Age, mean (SD)	9.96	(0.39)
Sex, n(% female)	2,337	(52.0%)
Ethnic group, n(%)		
white European	1,082	(24.1%)
South Asians	1,137	(25.3%)
black African-Caribbean	1,237	(27.5%)
Other	1,039	(23.1%)
NS-SEC group, n(%)		
Managerial & professional occupations	1,254	(27.9%)
Intermediate occupations	1,154	(25.7%)
Routine & manual occupations	1,374	(30.6%)
Inactive	462	(10.3%)
Unclassified / Missing	251	(5.6%)
Month of measurement, n(%)		
January	547	(12.2%)
February	435	(9.7%)
March	373	(8.3%)
April	281	(6.3%)
May	437	(9.7%)
June	454	(10.1%)
July	323	(7.2%)
September	309	(6.9%)
October	391	(8.7%)
November	615	(13.7%)
December	330	(7.3%)
